# Effect of Airflow Exposure on the Tear Meniscus

**DOI:** 10.1155/2012/983182

**Published:** 2012-04-10

**Authors:** Shizuka Koh, Cynthia Tung, Ranjini Kottaiyan, James Zavislan, Geunyoung Yoon, James Aquavella

**Affiliations:** ^1^Flaum Eye Institute, University of Rochester, Rochester, NY 14642, USA; ^2^Department of Ophthalmology, Osaka University Graduate School of Medicine, Osaka 565-0871, Japan; ^3^The Institute of Optics, University of Rochester, Rochester, NY 14642, USA

## Abstract

*Purpose*. To compare the effect of airflow exposure on the tear meniscus and blink frequency in normal and evaporative dry eye subjects. *Methods.* In 9 normal subjects and 9 short tear breakup time (SBUT) dry eye subjects, lower tear meniscus height (TMH) and area (TMA) and blink frequency were measured with anterior segment optical coherence tomography (OCT) before and after 5 minutes of airflow exposure (1.5 ± 0.5 m/s). *Results.* In SBUT dry eyes, both TMH and TMA decreased significantly (*P* = 0.027, *P* = 0.027) with a significant increase of blink frequency after airflow exposure, while significant increase in TMA was found in normal eyes. *Conclusion*. Measurement of the tear meniscus with anterior segment OCT seems to be useful as a noninvasive and objective method for evaluating the effect of airflow on tear film.

## 1. Introduction

Environmental factors have long been known to influence the precorneal tear film. Under environmental stress, such as low relative humidity or high air velocity, faster evaporation rates and thinning of the tear film predispose the cornea to dry spot formation, which may lead to dry eye symptoms and changes in the corneal epithelium [1–4]. The blinking condition is reported to be altered as a result of the changes on the ocular surface [1, 5, 6].

High air velocity causes evaporation of water from the precorneal tear film by eliminating the boundary of air adjacent to the tear film in conditions of stagnant ambient air. Wyon and Wyon [2] showed that exposure of the tear film to high air velocity (1.0 m/s) for 30 minutes caused a significant decrease in tear stability as measured by tear film breakup time (BUT) in healthy eyes. However, exposure to air velocity of 0.5 m/s for 30 minutes showed no significant differences. Nakamori et al. also reported that high air velocity (1.4 m/s) is associated with an increase (16.9 ± 2.9 to 22.8 ± 4.0) in blink frequency in normal eyes [6].

Recently, controlled-environment chambers or controlled adverse environment settings have been used to precisely regulate temperature and humidity in the evaluation of the tear film [7, 8]. A previous study reported the effect of temperature and humidity, using controlled-environment chambers [8]. However, to our knowledge, there has been no report studying airflow as an isolated variable using controlled-environment chambers.

Anterior segment optical coherence tomography (OCT) has been used widely to image the anterior segment noninvasively [9]. Previous studies [10–14] have reported that evaluation of the tear film using OCT allows us to quantify tear meniscus dimensions.

Therefore, we conducted the current study to investigate the changes in lower tear meniscus dimensions after exposure to airflow by means of using OCT and to understand the influence of airflow on the tear film volume dynamics, specifically, in evaporative dry eyes in comparison with normal eyes.

## 2. Methods

This study was approved by the institutional review board of University of Rochester. The research followed the tenets of the Declaration of Helsinki. Informed consent was obtained from each subject after the potential consequences of the study were explained fully. 

### 2.1. Subjects

Nine eyes of 9 normal volunteers (3 women, 6 men; average age 25.0 ± 1.9 years) and nine eyes of 9 short tear film breakup time dry eye (SBUT dry eye) patients (4 women, 5 men; average age 29.8 ± 8.2 years) were enrolled in this study at the Flaum Eye Institute, University of Rochester. The inclusion criteria for the SBUT dry eye group were as follows [15, 16] a tear film breakup time shorter than 5 seconds (average of 3 values evaluated with fluorescein), dry eye symptoms, absence of fluorescein staining of the ocular surface, and Schirmer I values that showed no tear deficiency (5 minutes without anesthesia) (21.0 ± 9.8 mm). Between two groups, there was no significant difference in age or Schirmer test values. BUT was significantly decreased in the SBUT dry eye group (3.9 ± 0.9 sec) when compared with the normal group (8.2 ± 1.4 sec). (*P* < 0.001, *t*-test) The exclusion criteria for both the normal and SBUT dry eye groups included other ocular disease or previous ocular surgery, systemic disease, or a history of drug use that would alter the ocular surface. The right eye of each subject was used for each measurement. To avoid the effects of other tests on the tear meniscus evaluation, tear function tests and slit-lamp examinations were conducted on a separate day before the study measurements.

### 2.2. Measurements

Subjects were evaluated in a controlled-environment chamber which was used to precisely regulate temperature, humidity, and airflow. Temperature and humidity were maintained at 22 ± 1°C and 40 ± 2%.

A commercial anterior segment OCT (Visante, Zeiss, Meditec, Inc., Dublin, CA) was used to make noninvasive and objective measurements of the tear film meniscus dimensions. It uses a superluminescent diode light source at wavelength of 1310 nm. The axial resolution was 18 *μ*m, and the transverse resolution was 60 *μ*m. Cross-sectional images of the lower tear meniscus were taken vertically across the central cornea using OCT, and the images were recorded continuously with desktop screen capture software (AmaRecCo version 1.21) at 2 frames per second. The lower tear meniscus was chosen for analysis in this study, as it is reported to be useful in evaluating tear volume on the ocular surface [17, 18]. OCT recordings were performed twice for each subject; once without airflow (baseline) and once after 5 minutes of airflow exposure. During the measurement, subjects were asked to rest their head on the chin rest of the OCT and instructed to gaze at the built-in target of the OCT with involuntary blinks. Air was blown across the eye from the right side at a speed of 1.5 m/s perpendicular to the gaze, at a distance of 15 cm from the eye. The air was delivered from a pipe built into the wall of the controlled-environment chamber and was monitored with an anemometer. Blink frequency was determined by counting involuntary blinks recorded on the OCT over 1 minute.

### 2.3. Data Analysis

**Figure 1:**
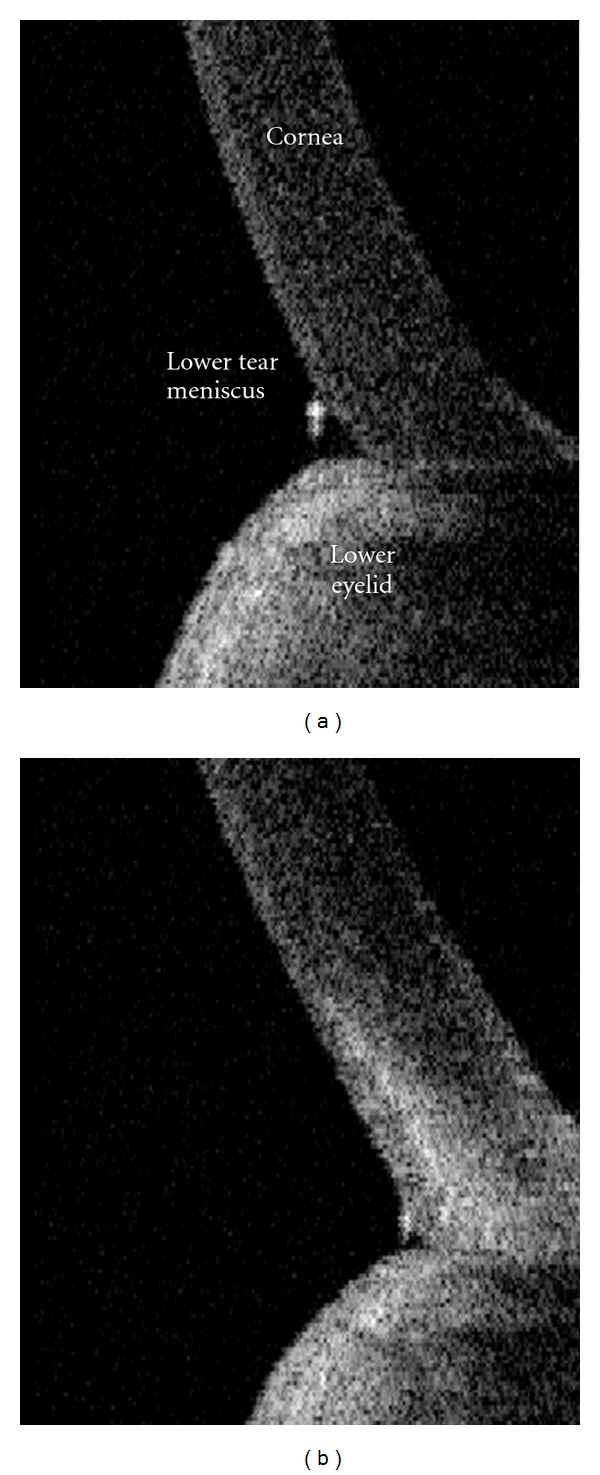
For each subject, a series of vertical cross-sectional images of the lower tear meniscus were taken with OCT. Analysis was done of the height and area of the triangular region at the junction of the cornea and lower lid. Representative OCT images (a) before and (b) after airflow exposure.

For each time point, OCT data was measured over 15 seconds. The frames recorded during blinks were not analyzed. In accordance with a previous study [14], all the OCT images were processed by a single trained observer using custom software. Lower tear meniscus height (TMH) and area (TMA) were calculated from cross-sectional OCT images of the lower tear meniscus (Figure 1). For each time point, average TMH and TMA were calculated over a 15-second interval and blink frequency was determined by counting the number of blinks recorded on the OCT over 1 minute. The beginning of the 1-minute time span used to calculate blink frequency coincided with the beginning of the 15-second interval used to calculate the average tear dimensions.

Data were analyzed using statistical analysis software JMP version 9 (SAS, Inc., Cary, NC). The Wilcoxon signed-rank test was used to compare TMH, TMA, and blink frequency before and after airflow exposure for each group. *P* < 0.05 was considered significant for all analyses.

## 3. Results

### 3.1. Changes in Tear Meniscus Dimensions with Airflow

**Figure 2:**
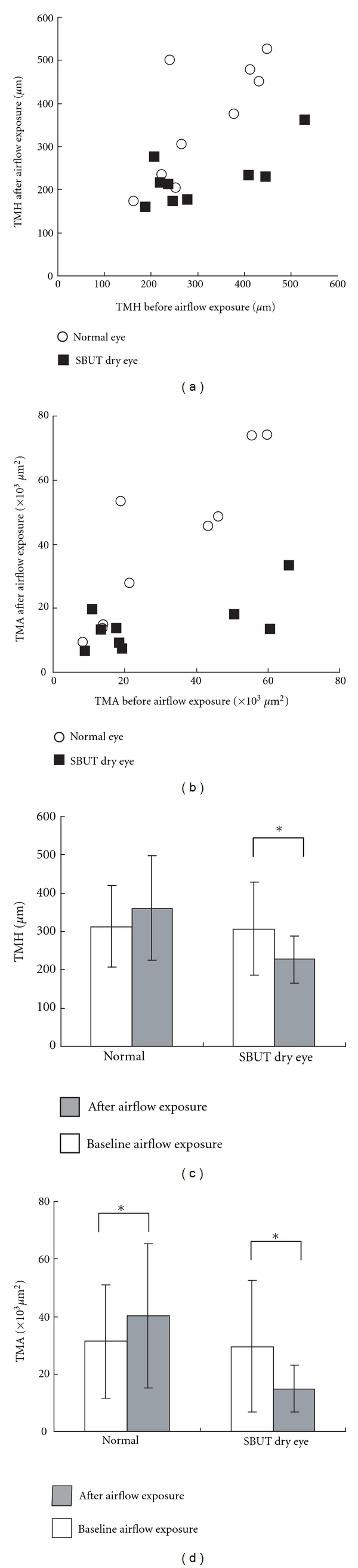
(a)-(b) Airflow-exposure-induced changes in the tear meniscus are presented for normal and short tear film breakup time dry eye (SBUT dry eye) groups. (c)-(d) In SBUT dry eye, both TMH and TMA decreased significantly (*P* = 0.027 and *P* = 0.027, resp., Wilcoxon signed-rank test). SBUT dry eye: short tear film breakup time dry eye; TMH: tear meniscus height; TMA: tear meniscus area.

Figure 2 shows the lower meniscus dimensions before and after exposure to 1.5 m/s airflow for 9 normal eyes and 9 SBUT dry eyes. In SBUT dry eye, there was a significant decrease in TMH of 80.87 *μ*m (*P* = 0.027) and a significant decrease in TMA of 14692.14 *μ*m^2^ (*P* = 0.027) after airflow exposure. In normal eyes, there was a nonsignificant increase in TMH of 47.67 *μ*m (*P* = 0.074) and in TMA of 8849.11 *μ*m^2^ (*P* < 0.001) after airflow exposure.

### 3.2. Changes in Blink Frequency with Airflow

**Figure 3:**
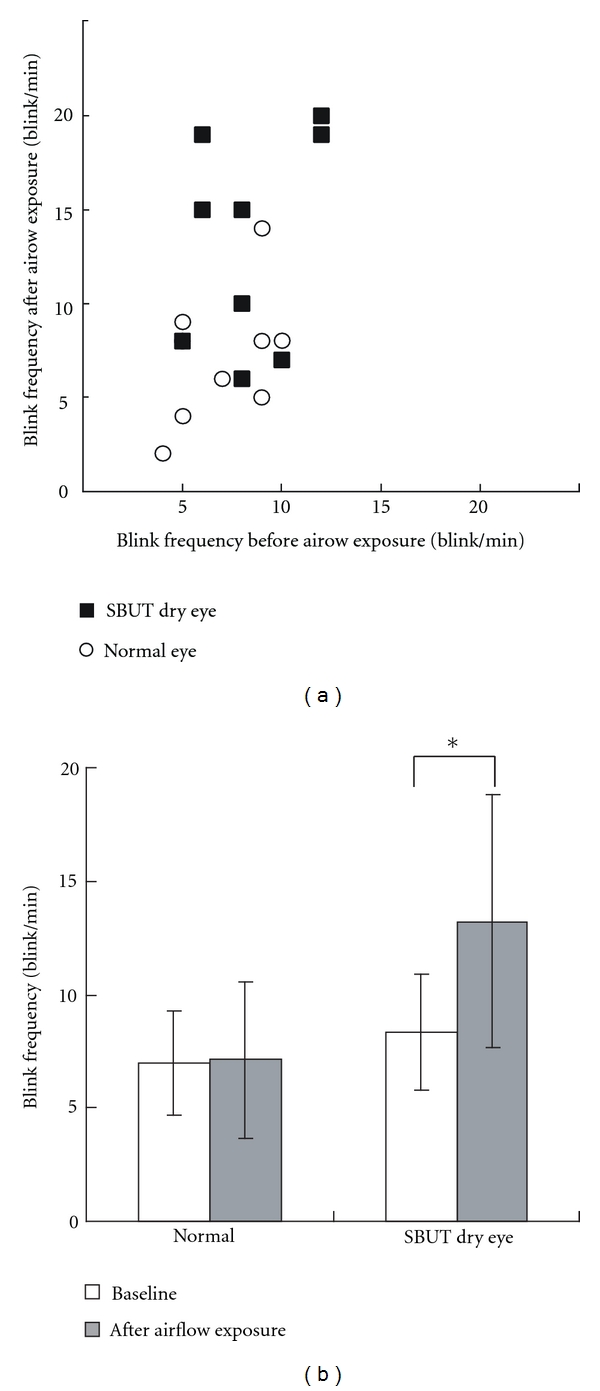
(a) Airflow-exposure-induced changes in blink frequencies are presented for normal and short tear film breakup time dry eye (SBUT dry eye) groups. (b) Changes in blink frequencies before and after airflow exposure are shown. Blink frequency increased significantly in SBUT dry eyes (*P* = 0.039, Wilcoxon signed-rank test), while there was no significant change in the blink frequencies of normal eyes. SBUT dry eye: short tear film breakup time dry eye.

Figure 3 shows the blink frequencies before and after exposure to airflow in both groups. There was no significant difference between the baseline blink frequency of normal eyes and SBUT dry eyes (*P* = 0.331, Wilcoxon signed-rank test). After airflow exposure, blink frequency increased significantly by 59% in SBUT dry eyes (*P* = 0.039), but no significant change was observed in the blink frequency of normal eyes (*P* = 0.917).

## 4. Discussion

In the current study, we found that lower tear meniscus dimensions significantly decreased and blink frequency significantly increased in SBUT dry eye after exposure to airflow of 1.5 m/s. In normal eyes, there was an increase in lower tear meniscus dimensions and little change in blink frequency.

Assuming that airflow exposure would make the tear film more prone to evaporation, a decrease in lower tear meniscus dimensions with the airflow exposure was expected in both groups. However, in the current study, it was found only in SBUT dry eyes, and no such pattern was found in normal eyes. According to the literature [19], in the initial stages of dry eye, it is considered that ocular surface damage results in reflex stimulation of the lacrimal gland. Reflex trigeminal activity is responsible for increased blink rate and compensatory lacrimal secretion. SBUT dry eye is a type of evaporative dry eye in which normal lacrimal tear secretion maintains normal tear volume, but tear stability is impaired. We speculate that, in normal eyes, the increase in tear meniscus volume caused by tear secretion through the reflex sensory loop [20] compensates adequately for the changes in tear film induced by airflow exposure, resulting in a net increase in tear meniscus dimensions. In contrast, a decrease in tear meniscus dimensions was seen in 8 out of the 9 SBUT dry eyes. In SBUT dry eye, we speculate that greater evaporation occurs in the setting of an unstable tear film by overriding the normal reflex tearing response, which results in a net reduction of tear volume in SBUT dry eyes compared with normal eyes, even though evaporation was not directly measured. Although we did not specifically evaluate the relationship between subjective symptoms and airflow, most of the SBUT dry eye subjects reported ocular discomfort with airflow exposure. Based on the results in the current study, some practical lifestyle modification advice for the SBUT dry eye patients are possible. Airflow control such as redirecting vents when using the air conditioner or using moisture chamber glasses [21] would be helpful in terms of keeping in moisture and reducing evaporation of tears by limiting airflow over the eyes.

We are assuming that normal eyes and SBUT dry eyes have nonpathologic reflex lacrimal production, based on the normal Schirmer I test conducted at screening. However, there may be a difference in the afferent arm of the reflex tearing response between the normal eyes and SBUT dry eyes. Normal eyes may be more efficient than SBUT dry eyes in producing reflex tearing to balance the increased evaporation observed in the setting of airflow exposure. It would be helpful to clarify the reflex tearing response in SBUT dry eye and aqueous tear-deficient dry eye in future studies.

Airflow exposure and evaporation of the tear film may stimulate the corneal blink reflex, resulting in greater blink frequency in SBUT dry eyes than in normal eyes. With regard to blink frequency in normal subjects, our findings are inconsistent with a previous study [6] that reported an increase in blink frequency with airflow exposure in healthy eyes. The disagreement between the results in the previous study [6] and the present study can be explained partly by the use of different measurement conditions. In the current study, the subjects were instructed to gaze at the built-in target of the OCT and blink freely, whereas, in the previous study, subjects were not asked to gaze at a target.

Previously, we used simultaneous measurements of ocular aberrations and lower tear meniscus dimensions to demonstrate that baseline tear meniscus just before the blink correlates with the initial postblink optical quality, especially in SBUT dry eye [14]. Although the blink rate used in the current study (involuntary blinking) was different from the blink rate used in the previous study (voluntary blinking every 6 seconds) [14], we can hypothesize that some SBUT dry eyes after airflow exposure would show greater degradation in optical quality during the initial postblink period because the tear meniscus dimensions are smaller. In the setting of an office environment, it is important to understand the relationship between airflow and ocular symptoms. The controlled-environment chamber allows independent modification of temperature, humidity, and airflow, facilitating the investigation of tear dynamics under many variations of everyday environmental conditions. Further studies that have greater sample size and include aqueous tear-deficient dry eye patients, using a controlled-environment chamber, will help to investigate the effect of different environmental conditions and further characterize the response of the tear film to different environmental stressors.

There are some limitations to this study. The images captured by our commercial time domain anterior segment OCT had limited resolution, so there was difficulty in detecting the tear film boundary in some images where the cross-points of the tear film, eyelid, and cornea were faint [14]. It will be important to use higher resolution OCT to make precise measurements of the tear film thickness. In the current study, we did not examine the repeatability of tear meniscus measurement with OCT. Although previous papers have showed the good repeatability in measuring the dimension of the tear meniscus [22, 23], we may need to know the repeatability with the OCT used in this study. The enrolled SBUT dry eyes were different from “typical dry eye” of aqueous tear-deficient dry eye, in a precise sense. [15, 16] There are many borderline cases that fall between evaporative dry eyes and healthy eyes, in which short TBUT and dry eye symptoms are found without ocular surface damage and tear deficiency. Moreover, the correlation between subjective symptoms and airflow-induced tear meniscus change would have been of help.

In conclusion, lower tear meniscus dimensions were observed to decrease and blink frequency was observed to increase in SBUT dry eye after exposure of 1.5 m/s airflow, while, in normal eyes, lower tear meniscus dimensions were observed to increase. Airflow exposure results in a decrease of tear meniscus dimensions in SBUT dry eyes, most likely due to the greater susceptibility of the tear film to evaporate in SBUT dry eye. Measurement of the tear meniscus with anterior segment OCT is useful as a noninvasive and objective method for evaluating tear film dynamics.
